# The Role of ^18^F-FDG PET/CT in Evaluating Elevated Levels of Tumor Markers in Breast Cancer

**DOI:** 10.4274/mirt.74436

**Published:** 2018-02-01

**Authors:** İnan Göktaş, Hakan Cayvarlı

**Affiliations:** 1 Ordu State Hospital, Clinic of Nuclear Medicine, Ordu, Turkey

**Keywords:** ^18^F-FDG PET/CT scan, breast cancer, biochemical tumor markers

## Abstract

**Objective:**

Our aim was to assess the diagnostic performance of integrated positron emission tomography/computed tomography (PET/CT) in the follow-up of breast cancer patients, who underwent a PET/CT scan due to a suspicion of recurrence based on elevated levels of serum tumor markers.

**Methods:**

Seventy-seven consecutive patients were included in this study. PET/CT scan results were compared with the final diagnoses that were obtained from histopathological sampling or a minimum 6 months of radiological follow-up. The sensitivity, specificity, positive predictive value (PPV), negative predictive value (NPV) and the diagnostic accuracy of PET/CT for detecting recurrence were calculated.

**Results:**

All 77 patients had increased serum cancer antigen 15-3 levels while 37 had increased serum carcinoembryonic antigen levels. According to PET/CT scan results, 59 of 77 patients (PET/CT positive) had local recurrence and/or distant metastasis while there was no pathological finding in 18 patients (PET/CT negative). In a follow-up of minimum 6 months, tumor recurrence was confirmed in 58 of “PET/CT positive” patients while no tumor recurrence was detected in 16 of “PET/CT negative” patients. According to these results the sensitivity, specificity, PPV, NPV and the diagnostic accuracy of PET/CT for detecting recurrence on a per-person basis were calculated as 98%, 88%, 96%, 94% and 96%, respectively.

**Conclusion:**

In case of elevated levels of serum tumor markers, PET/CT has a high diagnostic accuracy in detecting tumor recurrence in patients with breast cancer, and it is an effective modality that can be used in addition to conventional imaging techniques.

## Abstract (tr)

**Amaç:**

Bu çalışmada meme kanseri tanısı ile takip edilmekte iken tümör belirteçlerinde [kanser antijeni (CA) 15-3 ve karsinoembriyonik antijen (CEA)] yükselme saptanan ve bu sebeple nüks şüphesiyle yeniden evreleme amacıyla ^18^F-FDG pozitron emisyon tomografi/bilgisayarlı tomografi (PET/BT) çekilen hastalarda PET/BT’nin tanısal etkinliği araştırıldı.

**Yöntem:**

Çalışmaya 77 hasta dahil edildi. Hastaların PET/BT sonuçları ile çalışma sonrası minimum 6 aylık takip döneminde elde edilen histopatolojik ve radyolojik veriler karşılaştırıldı. Bu sonuçlara göre PET/BT’nin duyarlılığı, özgüllüğü, pozitif prediktif değerleri (PPV) ve negatif prediktif değerleri (NPV) ile tanısal doğruluğu hesaplandı.

**Bulgular:**

Çalışmaya dahil edilen hastaların tamamında CA 15-3 düzeyi yüksek iken, CEA düzeyi 37 hastada yüksek idi. PET/BT sonuçlarına göre 77 hastanın 59 tanesinde lokal nüks ve/veya uzak metastaz şüpheli lezyon saptanırken, 18 hastada herhangi bir patolojik bulguya rastlanmadı. Minimum 6 aylık takip neticesinde PET/BT’de şüpheli lezyon saptanan 59 hastanın 58 tanesinde lokal nüks ve/veya uzak metastaz varlığı doğrulanırken, PET/BT’de patolojik bulgu saptanmayan 18 hastanın 16 tanesinde takipleri süresince lokal nüks ve/veya uzak metastaz gelişmedi. Bu sonuçlara göre çalışmamızda PET/BT’nin hasta bazında duyarlılığı %96, özgüllüğü %94, PPV %98, NPV %88 ve tanısal doğruluğu %96 olarak bulundu.

**Sonuç:**

^18^F-FDG PET/BT, meme kanseri tanısı ile takip edilmekte iken tümör belirteç yüksekliği saptanan hastalarda lokal nüks ve/veya uzak metastazın araştırılması için tanısal etkinliği yüksek non-invaziv ve konvansiyonel görüntüleme yöntemlerine ek olarak kullanılabilecek etkili bir görüntüleme yöntemidir.

## INTRODUCTION

Breast cancer is the most common cancer among females. It is also the leading cause of cancer related death among females worldwide, with an estimated 1,7 million new cases and 521.900 deaths in 2012 (1).

Recurrence in breast cancer can occur even after 15 years following primary therapy, thus requiring life-long routine follow-up (2). Early detection of tumor recurrence improves long-term survival rates as well as quality of life.

Cancer antigen 15-3 (CA 15-3) and carcinoembryonic antigen (CEA) are two frequently used tumor markers in the follow-up of breast cancer (3,4). However, the results of many studies about these tumor markers in follow-up of breast cancer are inconsistent, even conflicting with each other. Previous studies have been conducted to quantitatively evaluate the serum levels of these two tumor markers and some found no significant correlation between increased tumor marker levels and recurrence while some found a significant correlation between them (5,6,7).

Although the definitive diagnostic method of breast cancer recurrence is histopathologic confirmation, it is not always easy to perform due to deep location or being very small in size or being too close to organs or great vessels making sampling either difficult or risky. Morphological imaging studies or tumor markers can be used to evaluate breast cancer recurrence. Currently, the most commonly used morphological imaging methods for detecting breast cancer recurrence include mammography, ultrasound (US), computed tomography (CT) and magnetic resonance imaging (MRI). However, both tumor markers and morphological imaging studies have some limitations. For instance, tumor markers can neither localize the recurrence site nor differentiate loco-regional recurrence from distant metastasis. Even though morphological imaging studies can detect both loco-regional recurrence and distant metastasis, it is not always easy to discriminate post-operational changes from loco-regional recurrence. In addition, they also cannot evaluate the viability of the tumor tissue or small lesions since their diagnostic criteria depends on size measurement and morphological changes.

Integrated positron emission tomography (PET)/CT scan is a functional imaging modality that can measure increased glucose metabolism in tumor cells by using ^18^F-fluorodeoxyglucose (^18^F-FDG) as a radiopharmaceutical agent. Recently this imaging modality is also frequently performed for evaluating breast cancer, like many other types of cancers (8,9). However, the data on the value of ^18^F-FDG PET/CT in evaluating breast cancer recurrence in case of elevated levels of tumor markers is limited and not clear (10).

In clinical practice, during the follow-up of breast cancer, it is difficult to manage treatment when serum tumor marker levels increase without any morphological imaging finding or when suspicious morphological imaging findings are found in terms of breast cancer recurrence but histopathological confirmation is not convenient. In such circumstances, PET/CT scan can be used for evaluating recurrence (11,12).

That is why, in this study we aimed to assess the diagnostic performance of ^18^F-FDG PET/CT in evaluating recurrence in case of elevated levels of serum tumor markers (CA 15-3 and CEA) during follow-up of breast cancer, and to find the optimal cut-off values of serum tumor markers that can be used in discrimination of tumor recurrence when reporting PET/CT.

## MATERIALS AND METHODS

Seventy-seven consecutive patients who were followed-up for breast cancer and who underwent ^18^F-FDG PET/CT scan due to suspicion of recurrence based on elevated levels of serum tumor markers (CA 15-3 and CEA) were included in this study. Elevated serum tumor marker levels were accepted as >25 U/mL for CA 15-3, and >3.8 ng/mL for CEA. PET/CT scan results were compared with the final diagnoses that were obtained from histopathologic sampling or at least 6 months of radiological follow-up.

### ^18^F-FDG PET/CT Imaging

All scans were performed by using an integrated PET/CT system (Discovery 600; GE Healthcare, Milwaukee, USA) that consisted of a full-ring high-resolution bismuth germanate oxide PET and a 16-slice CT. Patients were fasted for at least 6 hours before imaging. Blood glucose levels were checked to be less than 200 mg/dL before injection of 10 to 15 mCi of ^18^F-FDG. 500 mL of oral contrast was administered and intravenous injection of ^18^F-FDG was followed by a period of approximately 60 minutes. The images were obtained from the vertex of the head to mid-thigh.

### Image Analysis

Two experienced nuclear medicine physicians interpreted PET/CT images. The readers were blinded to the results of previous imaging studies and to the follow-up data. For the purpose of statistical analysis, patients who have at least one positive PET lesion compatible with recurrence on PET/CT were categorized as “PET/CT positive” and all others as “PET/CT negative”. Then, the PET/CT data were compared with the follow-up data. If a patient has both loco-regional recurrence and distant metastasis but the PET/CT detected only one component (i.e. PET/CT detected the loco-regional recurrence but not the distant metastasis or vice versa) then PET/CT scan result was classified as false negative. The golden standard in this study was either radiological follow-up (in most of the patients) or histopathologic confirmation. Radiologically confirmed recurrence was defined as detection of recurrence by conventional imaging modalities (mammography, US, CT or MRI) within 6 months of PET/CT scan. When a suspicious lesion showed interval increment in size during follow-up or interval decrement in size after radio/chemotherapy it was accepted as a radiologically confirmed recurrence. A patient was accepted as free of recurrence after a negative radiological follow-up within at least 6 months of PET/CT scan. Recurrence detected after this period was interpreted as a new recurrence.

### Statistical Analysis

A retrospective analysis of prospectively collected archive data was performed. The Statistical Package for Social Sciences version 22.0 (SPSS Inc,; Chicago, IL, USA) was used for statistical analysis. Tests with a p value less than 0.05 were considered as statistically significant. Patient based sensitivity, specificity, positive predictive value (PPV), negative predictive value (NPV) and diagnostic accuracy of PET/CT were calculated. Receiver operating characteristic analysis was used to detect the optimal cut-off serum tumor marker levels that can be used in interpreting PET/CT. Kappa coefficient was used in the measurement of agreement analysis. Written informed consent was obtained from each patient included in this study.

## RESULTS

Seventy-seven consecutive patients were included in this study. The mean age of patients was 50.9, ranging from 27 to 78. Of all 77 patients, all of them had increased (>25 U/mL) serum CA 15-3 levels while 37 of them had increased (>3.8 ng/mL) serum CEA levels. In terms of histopathologic classifying; 66 patients had invasive ductal carcinoma, 6 had invasive lobular carcinoma and 5 had other type of tumors. According to TNM staging; 2 patients were classified as stage 1, 17 as stage 2, 24 as stage 3 and 34 had stage 4 disease. Patient characteristics are summarized in Table 1.

According to PET/CT scan results, 59 of 77 patients (PET/CT positive) had local recurrence and/or distant metastasis (57 were reported as having distant metastasis and 2 were reported as having loco-regional recurrence) while 18 were reported as having no pathological finding (PET/CT negative).

Bone metastasis was reported in 48 patients while liver metastasis was reported in 14, lung metastasis in 3, plural metastasis in 4, brain metastasis in 3, adrenal gland metastasis in 2, peritoneal metastasis in 1, spleen metastasis in 1, regional lymph node metastasis in 8, and extra-regional lymph node metastasis in 15 patients.

Of all 77 patients evaluated in this study; 58 of “PET/CT positive” patients and 2 of “PET/CT negative” patients were confirmed to have tumor recurrence, and 1 of “PET/CT positive” patients and 16 of “PET/CT negative” patients were accepted as negative for tumor recurrence finally. PET/CT scan results were false positive in 1 patient and false negative in 2 patients. In the follow-up, one patient who has been reported as having distant metastasis in PET/CT (false positive) was diagnosed as having mediastinal granulomatous disease finally. On the other hand, out of the 2 patients who have been reported as having no pathological findings in PET/CT (false negative) one was diagnosed with bone and pleural metastasis 5 months after the PET/CT scan, while bone metastasis in addition to neck and mediastinal lymph node metastasis was detected 12 months after the PET/CT scan in another patient.

Final diagnoses were obtained by histopathologic sampling in 23 (30%) of 77 patients and by radiological follow-up in the remaining 54 (70%). Histopathologic confirmation was obtained in 2 of 2 loco-regional recurrence site, 6 of 8 regional lymph node metastasis site, 6 of 15 extra-regional lymph node metastasis site, 4 of 48 bone metastasis site, 2 of 4 pleural metastasis site, 1 of 3 lung metastasis site and 2 of 14 liver metastasis site. Other sites of recurrence were confirmed by radiological follow-up. PET/CT scan results and correlation of PET/CT with final diagnosis are listed in Table 1.

Based on these results, on a per-person basis, there was a statistically significant correlation between PET/CT scan results and final diagnosis (kappa: 0.89, p=0.000 <0.05) with a sensitivity, specificity, PPV, NPV and diagnostic accuracy of 96%, 94%, 98%, 88% and 96% respectively.

There was no statistically significant correlation between elevated serum CA 15-3 levels (>25 U/mL) and final diagnosis (kappa: 0.0, p=1.0 >0.05). Nevertheless, a statistically significant correlation was detected if an optimal cut-off value of 40 U/mL was used to discriminate tumor recurrence when reporting a PET/CT scan (kappa: 0.35, p=0.001 <0.05) with a sensitivity, specificity, PPV and NPV of 76%, 64%, 88% and 44%, respectively. There was no statistically significant correlation between elevated serum CEA levels (>3.8 ng/mL) and final diagnosis (kappa: 0.16, p=0.081 >0.05). However, when reporting the PET/CT scan, if an optimal cut-off value of 4.8 ng/mL was used to differentiate tumor recurrence, a statistically significant correlation was detected (kappa: 0.21, p=0.017 <0.05) with a sensitivity, specificity, PPV and NPV of 50%, 82%, 90% and 31%, respectively.

## DISCUSSION

One of the major problems in breast cancer follow-up is detecting loco-regional recurrence and/or distant metastasis since the 80% 5-year survival rate in early disease is decreased to 15% in advanced stages, and since a considerable amount of patients are diagnosed at advanced stages. Moreover, the recurrence rate is very high -nearly 30% in early stage disease, and it can occur even 15 years after primary therapy. The 5- and 10-year recurrence rates after primary therapy are reported as 6 and 12% in stage 1-2 disease, respectively (2,13). Patients who have tumor recurrence occurring after primary therapy have a chance of curative treatment. Therefore, early detection of recurrence and restaging is important for planning the optimal treatment regimen and selecting the patients who can be curatively treated.

In the follow-up of breast cancer, an increase in tumor marker levels usually indicates recurrence, but its sensitivity is low and the sensitivity spreads in a wide range in different studies. An elevated tumor marker level is not always related to a recurrence. Moreover, tumor markers cannot localize the recurrence and cannot show if the disease is widespread or not.

In a study of Lumachi et al. (14), they found the sensitivity of CEA and CA 15-3 as 38.1% and 61.1% and the specificity of both tumor markers as 98.8% and 91.2%, respectively, in detecting breast cancer recurrence. In another study, Guadagni et al. (15) reported the sensitivity of CEA as 41.3% and the sensitivity of CA 15-3 as 80.8% in recurrent disease.

Some studies showed that the tumor marker levels increase before clinical or radiological findings of recurrence (16,17). In a study by Nicolini et al. (11), patients were divided into two groups; the first group of patients who received medical therapy in case of negative conventional imaging findings but significant increase in one or more components of CEA-TPA-CA 15-3 tumor marker panel (“tumor marker guided” treatment) and the second group of patients who were treated only after radiologically confirmed recurrence (conventional treatment). As a result of this study, “tumor marker guided” treatment prolonged disease-free and overall survival rates significantly (11).

Gallowitsch et al. (18) evaluated the role of ^18^F-FDG PET in the follow-up of breast cancer in case of clinical suspicion of recurrence and/or tumor marker increase in correlation with conventional imaging modalities and reported on the advantages of ^18^F-FDG PET in the diagnosis of metastases when compared with conventional imaging modalities. In patients with clinical suspicion of tumor recurrence but not increased tumor marker levels, ^18^F-FDG PET was found to be a reliable imaging modality for detecting recurrence (18).

In parallel with the aforementioned study, in the study investigating the diagnostic accuracy of FDG PET/CT, CT and bone scintigraphy in patients with suspected breast cancer recurrence, Hildebrandt et al. (19) found that PET/CT was accurate in detecting recurrence and ruling out distant metastasis. They suggested that PET/CT had higher accuracy as compared to conventional imaging modalities in this patient group (19).

In the study by Liu et al. (20) evaluating the impact of FDG PET on detecting breast cancer recurrence based on asymptomatically elevated tumor marker levels, FDG PET correctly detected recurrence in 35/38 sites in 25/28 patients, and they reported the sensitivity and accuracy of FDG PET as 96% and 90%, respectively (20). Similarly, Lonneux et al. (21) found that FDG PET detected recurrence in 37/39 sites in 31/33 patients in their study evaluating the role of ^18^F-FDG PET imaging in patients with a suspicion of breast cancer recurrence due to tumor marker increase, and they concluded that FDG PET is highly sensitive for detecting distant metastasis despite a low specificity (21). The higher specificity rate detected in our study as compared to these two studies can be attributed to the usefulness of CT integration to PET devices, making discrimination of degenerative changes or physiological uptakes from pathological ones easier. In addition, both the high number of patients and the lower levels of serum tumor markers in our study as compared to those of Liu et al. (20) further confirm the high efficacy of PET/CT, and increase the importance of our study.

PET/CT is a highly sensitive and effective modality for evaluating breast cancer recurrence in case of an increase in tumor marker levels in asymptomatic patients. However, patient management should not be based only on PET/CT results due to its low specificity, and additional radiologic or histopathologic confirmations are required.

In our study, all 77 patients had increased CA 15-3 levels. In 60 of these patient’s tumor recurrence was confirmed. According to this data, CA 15-3 had a PPV of 77%. In 37 patient’s, serum CEA levels were increased. Thirty-two of these patients had confirmed recurrent disease. Therefore, the PPV of CEA in detecting tumor recurrence was 86%. Nevertheless, it must be remembered that 37 patients had elevated levels of both serum CEA and CA 15-3, so high PPV of CEA is not an unexpected result if both serum tumor markers are used together.

In our study, PET/CT correctly detected 58 of 60 patients who had confirmed tumor recurrence, and 16 of 17 patients who were confirmed as negative for tumor recurrence yielding a diagnostic accuracy of 96%. We found that in case of elevated levels of serum tumor markers, PET/CT has a high diagnostic accuracy in detecting tumor recurrence in patients with breast cancer.

In conclusion, this study provides significant evidence about the value of PET/CT in evaluating breast cancer recurrence in case of elevated levels of serum tumor markers during follow-up. Moreover, our results demonstrate that PET/CT can allow optimization of the treatment planning and might be considered in clinical decision-making process.

### Study Limitations

There are several limitations to our study. First, histopathologic confirmation of recurrence was not provided in most cases. In addition, the majority of included patients had advanced staged disease that increased the possibility of recurrence.

Further well-designed clinical studies are required to analyze the value of PET/CT in evaluating breast cancer recurrence. Also, the role of PET/CT in different histological subgroups of breast cancer should be evaluated.

## CONCLUSION

^18^F-FDG PET/CT is a noninvasive imaging modality that enables whole body scanning at once. In case of elevated levels of serum tumor markers, ^18^F-FDG PET/CT has a high diagnostic accuracy in detecting breast cancer recurrence and it is an effective modality that can be used in addition to conventional imaging techniques.

## Figures and Tables

**Table 1:**
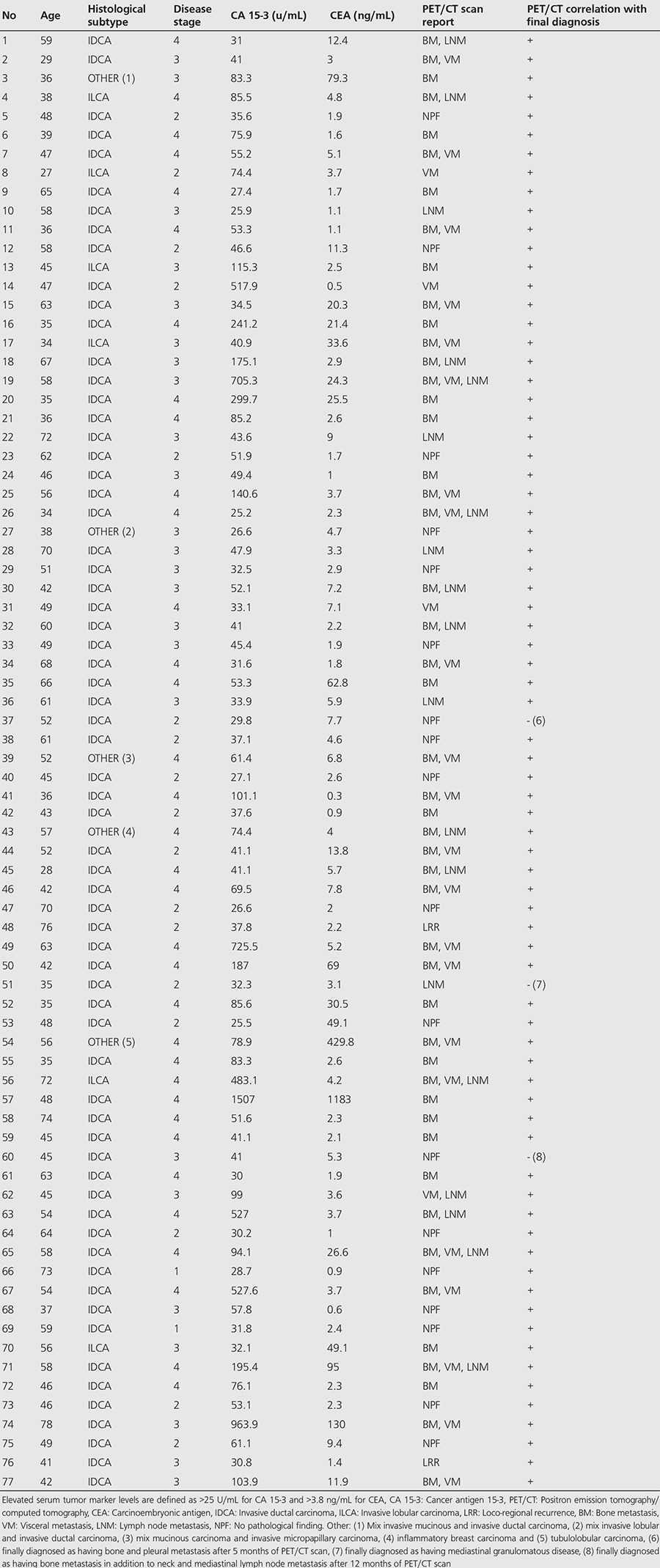
Patient characteristics and positron emission tomography/computed tomography scan results
